# Portable Fourier‐transform infrared spectroscopy and machine learning for sex determination in third instar *Chrysomya rufifacies* larvae

**DOI:** 10.1111/1556-4029.70054

**Published:** 2025-04-18

**Authors:** Aidan P. Holman, Davis N. Pickett, Hunter West, Aaron M. Tarone, Dmitry Kurouski

**Affiliations:** ^1^ Department of Biochemistry and Biophysics Texas A&M University College Station Texas USA; ^2^ Interdisciplinary Faculty of Toxicology Texas A&M University College Station Texas USA; ^3^ Department of Biology Texas A&M University College Station Texas USA; ^4^ Department of Entomology Texas A&M University College Station Texas USA

**Keywords:** forensic entomology, FTIR, infrared spectroscopy, maggots, sex

## Abstract

Forensic entomology is crucial in medicolegal investigations, utilizing insects—primarily flies—to estimate a supplemental post‐mortem interval based on their development at the (death) scene. This estimation can be influenced by extrinsic factors like temperature and humidity, as well as intrinsic factors such as species and sex. Previously, benchtop Fourier‐transform infrared (FTIR) spectroscopy coupled with machine learning demonstrated high accuracy in distinguishing the sex of third instar *Cochliomyia macellaria* larvae. This study leverages benchtop‐ and handheld‐based FTIR spectroscopy combined with machine learning models—Partial Least Squares Discriminant Analysis (PLSDA), eXtreme Gradient Boosting trees Discriminant Analysis (XGBDA), and Artificial Neural Networks Discriminant Analysis (ANNDA)—to differentiate between male and female *Chrysomya rufifacies* larvae, commonly found on human remains. Significant vibrational differences were detected in the mid‐infrared spectra of third instar *Ch. rufifacies* larvae, with a majority of peaks showing a higher abundance of proteins, lipids, and hydrocarbons in male larvae. PLSDA and ANNDA models developed using benchtop FTIR data achieved high external validation accuracies of approximately 90% and 94.5%, respectively, when tested with handheld FTIR data. This nondestructive approach offers the potential to refine supplemental post‐mortem interval estimations significantly, enhancing the accuracy of forensic analyses of entomological evidence.

**Highlights**

- Infrared spectroscopy enables differentiation between male and female *Chrysomya rufifacies* larvae.
- Differentiation is based on differences in proteins, lipids, and hydrocarbon vibrational bands.
- Chemometric analysis enabled over 90% accurate identification of the sex of *Ch. rufifacies* larvae.

## 1 INTRODUCTION

Forensic entomology is the study of insects and other arthropods in legal contexts, primarily to assist in determining the time of death during medicolegal investigations. This field leverages the life cycles of insects that colonize decomposing remains to estimate their time of colonization (TOC), which serves as a supplemental post‐mortem interval under specific assumptions [1]. Flies, being among the first insects to colonize human remains, play a central role in forensic analyses [2]. The development of these insects is influenced by external factors such as temperature and humidity and internal factors such as species and sex [3, 4]. Identifying these factors is critical for adjusting estimations, but determining the sex of fly larvae has posed significant challenges [5, 6].

Although limited, evidence increasingly suggests that sex plays a significant role in fly development, influencing age predictions and impacting forensic analyses. For instance, Noblesse et al. (2022) observed that male *Lucilia sericata* flies developed approximately 12 h faster than females, highlighting sex‐specific differences in developmental timelines [7]. Similarly, Pimsler et al. (2021) investigated sexually dimorphic patterns of gene expression in *Chrysomya rufifacies* (Macquart) throughout its development [8]. This study revealed male‐biased gene upregulation during larval stages, faster development—approximately 9 h quicker than females at 30°C—and larger body sizes in males.

Additional studies on Calliphorid flies, including *Chrysomya megacephala* and *Cochliomyia macellaria*, suggest similar trends where males develop faster under controlled conditions due to genetic and environmental factors, such as temperature and nutrient availability [9, 10]. These differences in development rates not only impact forensic age estimation but also emphasize the need for sex determination in immature stages to refine predictive models. Despite these insights, gaps remain in understanding how widespread these developmental differences are among forensically significant fly species. Moreover, the statistical effect size of these differences in forensic contexts has yet to be fully quantified.

One species in particular, *Ch. rufifacies*, is distinctive among Calliphoridae species due to its unique combination of developmental traits and ecological behaviors. Unlike many other flies, *Ch*. *rufifacies* exhibits a monogenic sex‐determination system, controlled by a dominant maternal‐effect gene (F′) [11]. This system results in single‐sex clutches. Furthermore, these flies are known for their predatory larval behavior, preying on other larvae at the same food source [12]. This behavior reduces competition and can delay or eliminate colonization timelines, which is vital in forensic entomology when assessing post‐mortem intervals (PMIs) [13]. For instance, Sanford (2014) observed *Ch*. *rufifacies* completely dominating the infestation of one individual, relegating other species, such as *L. eximia*, to the area surrounding the body [13, 14].

Sex‐specific developmental rates and behavioral differences in dispersal or predation could significantly influence TOC estimates and PMI calculations. Understanding these factors can improve forensic models and enhance the accuracy of medicolegal investigations. Determining the sex of immature stages, therefore, becomes crucial for addressing these questions and improving the accuracy of forensic entomological models.

Traditionally, adult flies are sexed using large‐scale morphological keys, which lack methods for distinguishing the sexes of larvae due to the absence of visible sexual dimorphism at this stage [15]. Molecular techniques, such as the PCR‐based methods developed by Jonika et al. (2020), have been used to determine the sex of adult and larval blowflies, including *Ch. rufifacies* and *Co. macellaria* [16]. However, these methods are destructive, rendering larval evidence unsuitable for further testing, and RNA can be unstable, potentially limiting its forensic application (though Smith (2024) reports that it is possible to obtain *transformer* RNA from ethanol stored samples of *Co. macellaria*). Similarly, Picard et al. (2012) employed flow cytometry to determine the sex of third instar *Lucilia sericata* larvae by genome size, a technique that also requires destructive sampling [17, 18].

Infrared (IR) spectroscopy offers a promising alternative as a noninvasive, nondestructive analytical tool capable of identifying and quantifying molecular structures through their unique vibrational signatures. Specifically, IR detects the unique vibrational modes of molecular bonds, which absorb infrared radiation when there is a change in dipole moment during vibration. These changes occur most prominently in polar functional groups, such as carbonyl (C=O), amine (N‐H), and methyl/methylene (C‐H) stretches, commonly found in proteins, lipids, and hydrocarbons. In adult insects, including flies, males and females commonly exhibit distinct cuticular hydrocarbon (CHC) profiles that are linked to mating behavior, desiccation resistance, and sexual communication [19, 20]. This technique has gained traction in forensic entomology for differentiating fly species, diets, ages, and sexes [21, 22, 23, 24, 25, 26]. For instance, Barbosa et al. (2018) demonstrated that IR spectra could effectively distinguish between the sexes and species of adult flesh flies (Diptera: Sarcophagidae), highlighting its potential for forensic applications [24]. Previously, our group demonstrated that benchtop Fourier‐transform infrared (FTIR) spectroscopy, combined with machine learning analysis, could accurately predict the sex of *Co. macellaria* larvae with high accuracy [26]. The adaptability and high‐throughput capabilities of IR spectroscopy make it an innovative method for advancing forensic investigations.

The current study investigates the use of FTIR spectroscopy for the highly accurate differentiation of male and female *Ch. rufifacies* larvae, a species commonly encountered at crime scenes throughout North America [27]. We utilize three machine learning models, Partial Least Squares Discriminant Analysis (PLSDA), eXtreme Gradient Boosting trees Discriminant Analysis (XGBDA), and Artificial Neural Networks Discriminant Analysis (ANNDA), to analyze and classify IR spectra from the larvae based on sex. This approach has the potential to greatly improve forensic investigations by offering a rapid, on‐site, and nondestructive method for more accurately estimating supplemental post‐mortem intervals.

## 2 MATERIALS AND METHODS

### 2.1 Insect acquisition, rearing, and identification

Adult *Ch. rufifacies* were sourced from a laboratory colony at the Texas A&M University Forensic Laboratory for the Investigation of Entomological Science (FLIES Facility) in College Station, TX, USA. Colony maintenance followed the methods described in Rusch et al. (2019) [28]. Flies were housed in 30 × 30 × 30 cm Bugdorm mesh cages (BioQuip, Rancho Dominguez, CA) in a temperature‐controlled room (~22°C, 50% relative humidity, and a photoperiod of 14:10 [L:D] h). Water was provided in 200 mL glass mason jars with paper towel wicks secured by rubber bands, and the flies were fed ad libitum with a 2:1 diet of table sugar and powdered milk.

Four to 5 days after pupal emergence, adult flies were given beef blood meals every other day, totaling four meals over 8 days. Following the fourth blood meal, 25 individual female *Ch. rufifacies* were isolated into 50 mL conical centrifuge tubes, each provided with ~5 g of beef liver wrapped in a Kimwipe as an oviposition site. These females were left to oviposit over a 24‐h period. Due to the species' monogenic sex‐determination system, wherein individual females are genetically predisposed to produce only male or only female offspring, each clutch is expected to be unisexual. However, only a subset of the 25 isolated females produced egg clutches.

In Event 1, two egg clutches were successfully retrieved, each from a different female. These two clutches were treated as independent, unisexual replicates. Fourteen hatchlings from clutch one and ten from clutch two were retained for analysis to balance the need for sufficient sample size with practical limitations on acquisition time, while still allowing for assessment of inter‐sample variability. The sex of each clutch was determined retrospectively: After larvae developed into adults, the sex of the emerging flies was assessed based on eye morphology (holoptic males vs. dichoptic females), allowing us to confirm the sex identity of the original larval clutch. Clutch one yielded only females, and clutch two yielded only males.

A second experimental event, Event 2, was conducted to generate an additional batch of third instar larvae, aimed at expanding the model training dataset and assessing whether inter‐generational variation in larval spectra influences model performance. In Event 2, the same procedure was applied to isolate new females and their egg clutches from a different generation of flies sourced from the same facility. Five clutches were obtained from five different females this way. Again, the total amount of eggs was reduced to 25 total, with 5 larvae from each clutch. The sex of each clutch was again confirmed by allowing the remaining larvae to pupate and eclose into adults. Clutches one and three produced only males, while clutches two, four, and five produced only females.

### 2.2 Infrared spectroscopy

**FIGURE 1:**
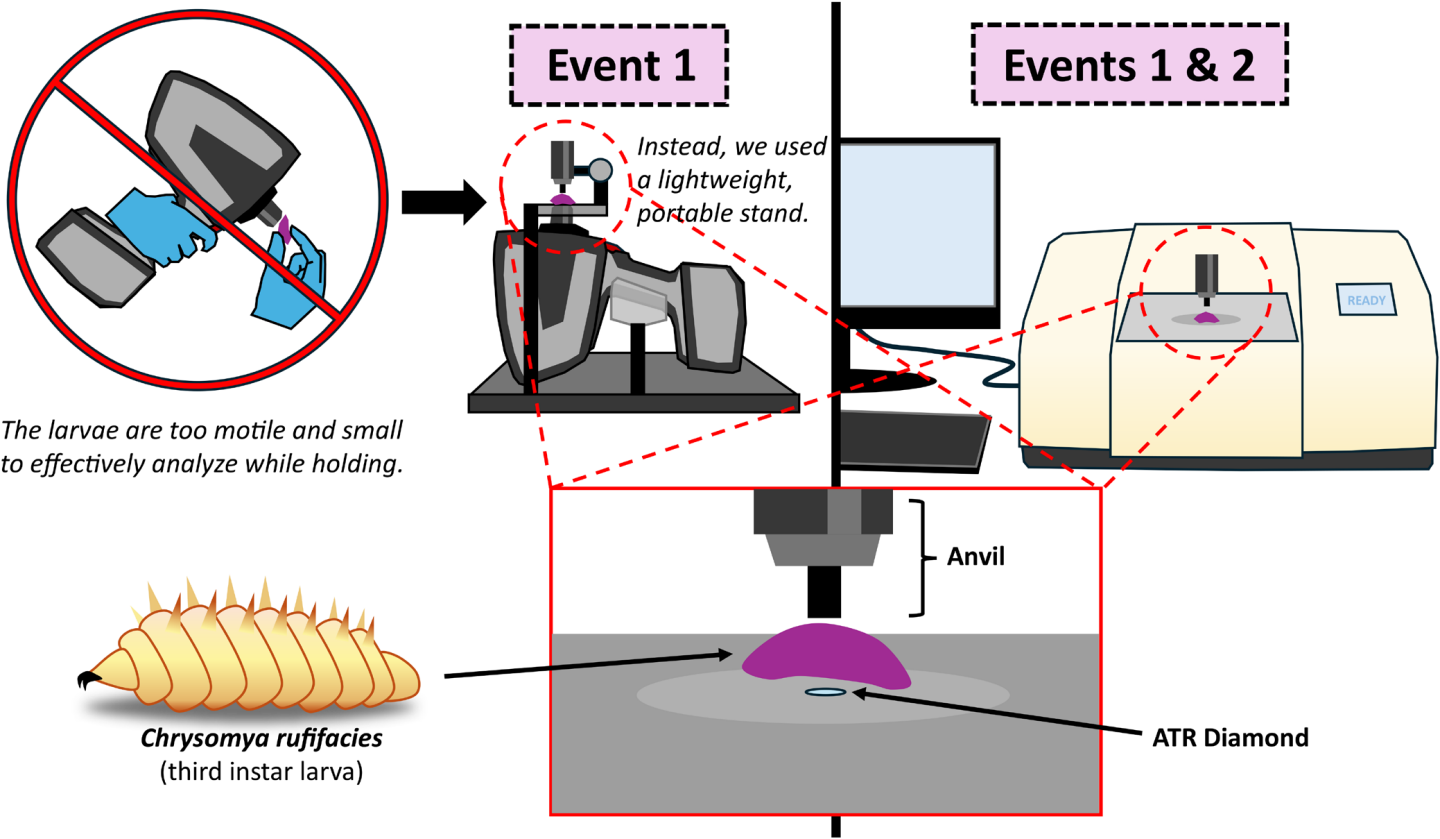
Position and analysis of larval samples per instrument.

Five attenuated total reflectance (ATR)‐corrected FTIR spectra were collected from the central region of each larva using either a Spectrum 100 IR spectrometer (*PerkinElmer, Inc*.) (benchtop) or 4300 Handheld FTIR system (*Agilent Technologies, Inc*.). To accomplish this, each larva was positioned with its ventral midsection directly over the ATR crystal on the instrument stage. Notably, the handheld FTIR device includes a lightweight portable stage, allowing it to function comparably to a benchtop system. The metal anvil was then gently lowered onto the dorsal midsection of the larva to stabilize it and ensure optimal contact with the crystal, thereby maximizing infrared signal transmission. The positioning and analysis setup are illustrated in Figure 1.

During Event 1, 22 larvae were analyzed using both benchtop and handheld FTIR systems, except for two larvae that were analyzed only with handheld FTIR (22 larvae, 110 total spectra) but were unintentionally killed during handling before benchtop FTIR analysis (20 larvae, 100 total spectra). In Event 2, 23 larvae were analyzed exclusively with benchtop FTIR, generating 115 total spectra.

Raw data were processed using attenuated total reflectance (ATR) correction (using a diamond composed of Zn/Se crystals) and displayed in absorbance by PerkinElmer spectrum express software (for benchtop FTIR) or Agilent MicroLab FTIR software (for handheld FTIR). Each spectrum was acquired from 650 to 4000 wavenumbers (cm^−1^) by combining 4 cumulative scans with 4 cm^−1^ resolution on the benchtop instrument and 30 cumulative scans with 4 cm^−1^ resolution on the handheld instrument. The applied force was 6 N for the benchtop.

### 2.3 Data analysis

All spectra were first trimmed from 900 to 3000 cm^−1^ to remove visually noisy regions across spectra. The trimmed spectra were then baseline‐corrected using automatic‐weighted least squares (2nd order) and smoothed using a Savitzky–Golay filter (2nd order, fl = 15 pt.) before analysis using MATLAB (*The MathWorks, Inc*.). Normalization was not required for the spectra from either instrument, as the laser power remains constant and immutable, and the system autonomously optimizes signal acquisition both before and during analysis. Additionally, a 1st derivative preprocessing (Savitzky–Golay) was applied in some instances to gauge model performance. Chemometric analysis of acquired spectra was done in MATLAB R2022b equipped with PLS_Toolbox 9.0 (*Eigenvector Research, Inc*.). Preprocessing of each model was done using the required mean centering and with or without 1st‐derivative Savitzky–Golay filtering (2nd order, fl = 15 pt.).

Internal validation refers to each model's 10‐fold cross‐validation. Briefly, this is done by dividing the dataset into 10 equally sized folds, training the model on 9 folds, testing it on the remaining fold, and repeating this process 10 times, with each fold serving as the test set once and averaging the results. External validation was done by testing each model, built on either Event 1 or Event 1 and 2 benchtop data, with the handheld IR data. External validation results were reported for all models, unless otherwise specified. Accuracy was calculated by dividing the number of correctly predicted instances by the total number of instances. Sample‐level accuracy was calculated using a majority‐vote rule where a sample is considered correctly predicted if more than 50% of its spectra were classified correctly, Tables S1–S18.

For the PLSDA model, the optimal number of latent variables was selected based on the lowest average classification error between the calibration and cross‐validation phases, considering only the first 10 latent variables to prevent overfitting. This selection process is illustrated in Figures S1 and S2. The chosen PLSDA model utilizes the specified number of latent variables (LVs)—which represent groups of characteristic variances in the data between sexes—to calibrate (build) the model. We then applied a 10‐fold cross‐validation to test the model's classification ability across all data.

For the XGBDA model, the optimal configuration is automatically determined by the algorithm, selecting the best combination of the number of trees (max_depth) and the learning rate (eta) based on performance metrics. These optimization results are shown in Figure S3. We then applied a 10‐fold cross‐validation on the calibrated data.

For the ANNDA model, we chose a neural network architecture with two hidden layers, consisting of 20 and 10 nodes in the first and second layers, respectively. The architecture was selected to balance complexity and predictive accuracy while avoiding overfitting. The model was trained using a backpropagation algorithm with a fixed learning rate and activation functions optimized for classification tasks. LVs were used to reduce dimensionality between classes and enhance feature extraction. We then applied a 10‐fold cross‐validation to assess the model's performance.

Model performance was evaluated using several standard classification metrics. Accuracy represents the proportion of all correctly classified instances, while sensitivity (or recall) measures the model's ability to correctly identify true positives for each class (e.g., correctly classifying male or female larvae). The global F1 score is the harmonic means of precision and recall across all predictions, offering a balanced performance metric, especially for imbalanced datasets. Matthew's correlation coefficient (MCC) provides a more comprehensive assessment by incorporating true and false positives and negatives; it ranges from −1 (complete misclassification) to +1 (perfect classification), with 0 indicating random prediction.

Finally, a Shapiro–Wilk test was performed in RStudio across all datasets and revealed that a majority of vibrational bands were not normally distributed. Thus, non‐parametric tests for differences in medians across vibrational bands were employed. Pairwise Wilcoxon rank‐sum tests (with a Bonferroni correction) and the Manhattan plot were generated in RStudio, using the “wilcox. test” and “ggplot2” R libraries.

## 3 RESULTS AND DISCUSSION

An FTIR spectrum can be divided into two main regions: the analytical region (≥1600 cm^−1^) and the fingerprint region (<1600 cm^−1^). The analytical region is useful for identifying functional groups with well‐defined characteristic peaks, such as O‐H (around 3200–3600 cm^−1^) and C‐H (2850–3300 cm^−1^) stretching vibrations. By contrast, the fingerprint region contains more complex molecular vibrations that depend on the overall chemical structure of the sample. This region is highly specific to individual compounds, making it useful for distinguishing between similar molecules. As shown in Table 1, several peaks in this region can correspond to multiple molecular vibrations and different biomolecules, requiring careful interpretation based on the sample's composition.

**TABLE 1:** FTIR bands and respective molecular vibrational modes and biomolecular assignments.

IR region	Wavenumbers (cm^−1^)	Peak assignment
Fingerprint	1053–1072	C‐O stretching; proteins [29, 30]
	1152–1159	C‐O stretching; lipids [29, 30, 31]
	1237	(Amide III) C‐N stretching, N‐H bending; proteins [29, 32, 33]
	1312–1314	O=C‐O bending; proteins [29, 34]
	1340	C‐O stretching; proteins and lipids [29, 30]
	1396–1400	C‐H stretching, C‐O bending; hydrocarbons, lipids, and proteins [29, 30]
	1450–1457	C‐H stretching, C=C stretching; hydrocarbons, lipids, and proteins [29, 30, 32]
	1539–1541	(Amide II) N‐H bending, C‐N stretching; proteins [29, 30]
	1631	(Amide I) C=O stretching, C‐N stretching, N‐H bending; proteins [22, 29, 30]
Analytical	2324	(R‐SO_2_)‐O‐H stretching; decomposition byproducts [29]
	2364	(R‐SO_2_)‐O‐H stretching; decomposition byproducts [29]
	2851	C‐H stretching; hydrocarbons [29, 35]
	2919–2920	C‐H stretching; hydrocarbons [29, 35]

**FIGURE 2:**
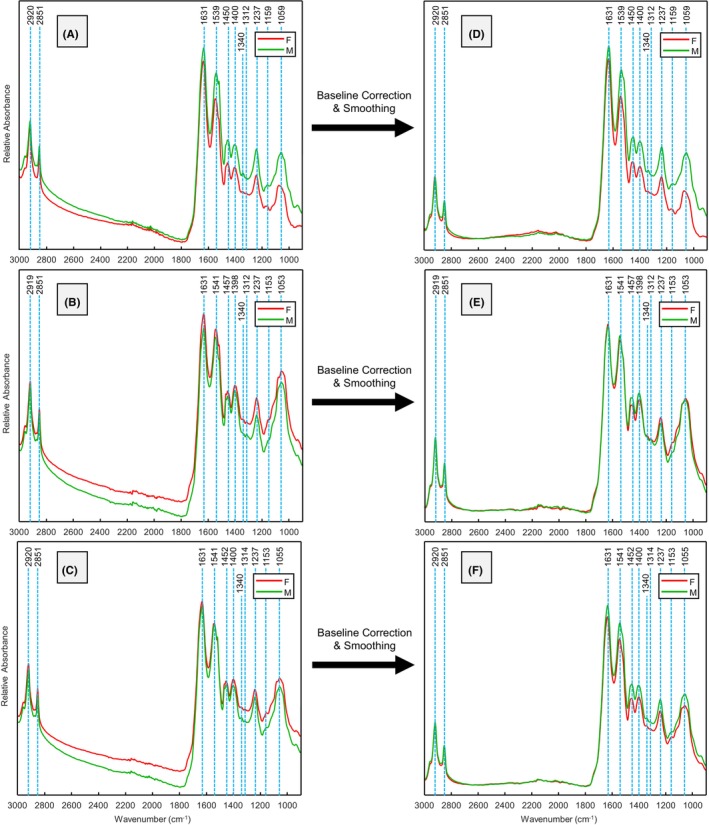
Mean benchtop FTIR spectra of male (green) and female (red) third instar *Ch. rufifacies* larvae analyzed in Event 1 (A), Event 2 (B), combined Events 1 and 2 (C), and after preprocessing (D–F).

During the first analysis event (Event 1), male and female third instar *Ch. rufifacies* larvae exhibited shared peaks at 1059, 1159, 1237, 1312, 1340, 1400, 1450, 1539, and 1631 cm^−1^ in the fingerprint mid‐IR region and at 2851 and 2920 cm^−1^ in the analytical mid‐IR region (Figure 2A,D). These peaks are primarily attributed to the sclerotin protein (and amino acids), wax‐lipid compositions, and other hydrocarbons of the larval cuticle (Table 1). During Event 2, slight shifts were observed in several vibrational bands, with both sexes sharing peaks at 1053, 1153, 1237, 1312, 1340, 1398, 1457, 1541, 1631, 2851, and 2919 cm^−1^ (Figure 2B,E). When the spectra from both sexes were combined, the mutable peaks converged near the center of their ranges, resulting in shared peaks at 1055, 1153, 1237, 1314, 1340, 1400, 1452, 1541, 1631, 2851, and 2920 cm^−1^ (Figure 2C,F). The mean and standard deviations for all benchtop and handheld male and female larval FTIR spectra can be found in Figure S4.

**FIGURE 3:**
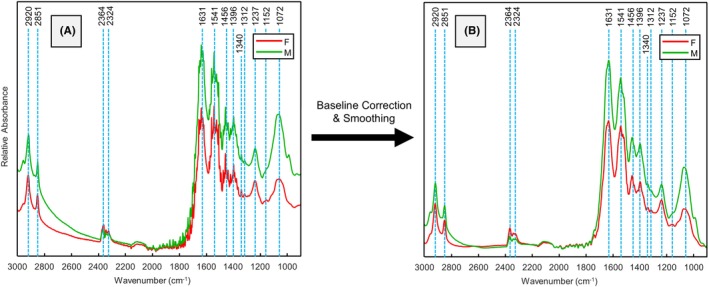
Mean handheld FTIR spectra of male (green) and female (red) third instar *Ch. rufifacies* larvae analyzed during event 1 before (A) and after preprocessing (B).

During Event 1, the same flies analyzed with benchtop FTIR were also measured using handheld FTIR, except for two flies that were analyzed with handheld FTIR but were killed during handling before benchtop analysis. Male and female third instar *Ch. rufifacies* larvae analyzed in this manner shared peaks at 1072, 1152, 1237, 1312, 1340, 1396, 1456, 1541, and 1631 cm^−1^ in the fingerprint mid‐IR region. Additionally, compared with the benchtop data, the handheld FTIR spectra showed two extra peaks in the analytical region, with shared peaks at 2324, 2364, 2851, and 2920 cm^−1^ (Figure 3). These “new” peaks (2324 and 2364 cm^−1^) are likely attributed to the decomposition byproducts from the larvae's meal (Table 1), as the larvae were first analyzed with the handheld FTIR and then with the benchtop FTIR during Event 1.

**FIGURE 4:**
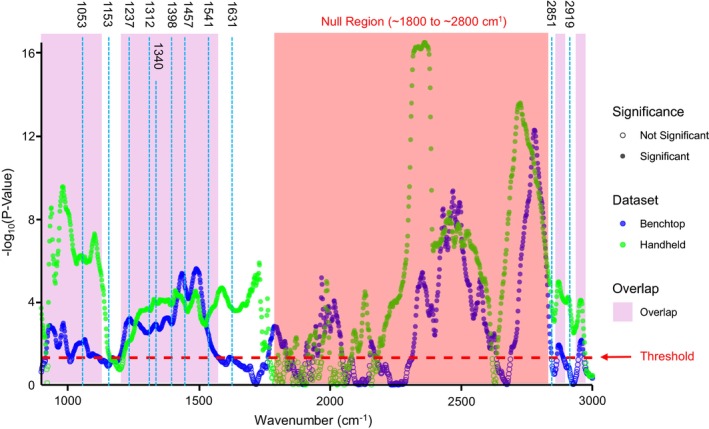
Manhattan plot for Wilcoxon test (with Bonferroni correction) results between male and female benchtop (blue) and handheld (green) FTIR spectra. The threshold corresponds to a *p*‐value of 0.05. Overlapping regions above the threshold were shaded in purple to demonstrate which FTIR bands were significant across both datasets. The “null region” refers to the ~1800–2800 cm^−1^ range in the FTIR spectra, which, despite the handheld IR data, lacks distinctive or biologically relevant peaks and exhibits minimal spectral variability. As such, any statistically significant differences observed in this region are unlikely to reflect meaningful biochemical variation and are more likely artifacts of baseline noise or low‐amplitude fluctuations.

Pairwise Wilcoxon rank‐sum tests were conducted across the combined benchtop and handheld datasets to compare instrumental variances between sexes, as shown in Figure 4. The two additional peaks identified in the handheld FTIR analysis were excluded from this comparison due to their absence in the benchtop FTIR data, referred to as the “null region.” The results indicate that most vibrational bands within the fingerprint region exhibit significant differences in median FTIR signals between male and female *Ch. rufifacies* across both datasets. Specifically, significant differences were observed at 1053, 1237, 1312, 1340, 1396, 1457, and 1541 cm^−1^, corresponding to various molecular vibrations of proteins, lipids, and other hydrocarbons. Pairwise comparison plots, generated in MATLAB (Figures S5 and S6), were used to assess trends in the relative abundance of these molecules between sexes. The analysis revealed that male third instar *Ch. rufifacies* consistently exhibited higher abundances of proteins, lipids, and hydrocarbons at all peaks of significance (Table 2).

**TABLE 2:** Pairwise comparison Wilcoxon test results for overlapping peaks between the combined benchtop (BT) and handheld (HH) datasets.

Wavenumbers (cm^−1^)	BT *p*‐value	HH *p*‐value	Higher ranks (M/F)?	Peak assignment
1053–1072	0.0097	*p* < 0.001	M	Proteins
1237	*p* < 0.001	0.0069	M	Proteins
1312–1314	0.0026	*p* < 0.001	M	Proteins
1340	0.0015	*p* < 0.001	M	Proteins/lipids
1396	0.0011	*p* < 0.001	M	Proteins/lipids/hydrocarbons
1450–1457	*p* < 0.001	*p* < 0.001	M	Proteins/lipids/hydrocarbons
1539–1541	0.0074	*p* < 0.001	M	Proteins

Currently, there are no studies that specifically identify the proteins, lipids, or hydrocarbons in the larval cuticles of *Ch. rufifacies* or many other fly species. However, research has explored differences in hydrocarbons and their concentrations between male and female flies, as observed in peaks at 1396 and 1450–1457 cm^−1^. For instance, Butterworth et al. (2020a) analyzed the cuticular hydrocarbons of *Ch. rufifacies* larvae using gas chromatography–mass spectrometry (GC–MS) and found distinct profiles between males and females [20]. The most abundant hydrocarbons identified were monomethylalkanes, n‐alkanes, and n‐alkenes. Further analysis revealed that males had a higher abundance of cuticular hydrocarbons in most blowfly species, with *Ch. rufifacies* males showing higher levels in six out of ten hydrocarbons analyzed, including nearly 28 times more C27:2 hydrocarbons than females. By contrast, females surpassed males in only four out of ten hydrocarbons, with no more than four times the abundance in those cases (Table 3).

**TABLE 3:** *Ch. rufifacies* GC–MS cuticular hydrocarbon analysis between sexes. Adapted from Butterworth et al. (2020) [20].

Hydrocarbon	Male relative abundance (%)	Female relative abundance (%)	M:F ratio
C21	0.15	0.077	1.9
C25:1	0.042	0.15	0.3
C27:2	0.12	0.0043	27.9
11‐Me‐C27	1.5	5.9	0.3
3‐Me‐C27	1.1	0.27	4.1
C29:1	2.9	9.7	0.3
(Unknown)	2.5	0.81	3.1
11,13‐Me‐C31	4.7	10	0.5
2‐Me‐C32	5.8	0.78	7.4
C33:1	5.7	1.3	4.4

Furthermore, evidence of protandry, a pattern where males develop or mature earlier than females, is increasingly documented across blowfly species. Notably, the same group, Butterworth et al. (2020b), demonstrated that in *Chrysomya varipes*, a close relative of *Ch. rufifacies*, male CHC profiles develop 1–2 days earlier than females post‐eclosion, coinciding with earlier reproductive maturity [19]. This developmental lead in males aligns with our findings of sex‐specific chemical signatures already present at the third instar larval stage in *Ch. rufifacies*, suggesting that sex‐linked differences in CHC composition may begin well before adult emergence, despite the shedding of the cuticle at each new life stage. These converging lines of evidence reinforce the biological plausibility of using vibrational spectroscopy to differentiate sexes in immature stages.

Other research on the sex‐dependent growth of *Drosophila* larvae has demonstrated that males tend to focus on increasing size during development, resulting in higher protein expression, while females often exhibit greater lipid storage, reflecting their reproductive needs later in life [36]. Although our results suggest that males might possess higher overall levels of proteins and lipids compared with females, the observed higher peak indices in FTIR spectra could be attributed to differences in the molecular composition or density of specific biomolecules, such as cuticular hydrocarbons or other surface lipids, which vary between sexes. These findings align closely with results from our previous study, where male *Co*. *macellaria* third instar larvae exhibited higher relative absorbances at most peaks in the fingerprint region, except for specific peaks at 1238 and 1547 cm^−1^, where females showed higher absorbance, and 1454 cm^−1^, which showed no significant difference [26].

In addition to the numerous significant differences in molecular abundance across peaks, which illustrate distinct spectra between male and female third instar *Ch. rufifacies* larvae, various machine learning models were employed to classify male and female spectra for forensic purposes. Two versions of each model were developed: one using only mean centering and the other incorporating a first‐derivative Savitzky–Golay filter followed by mean centering.

To evaluate the effectiveness of using a single dataset for calibration, as done previously [26], Event 1 benchtop FTIR data were used to calibrate PLSDA, XGBDA, and ANNDA models, which were then tested on Event 2 benchtop FTIR data (Table 4). The models with mean centering consistently outperformed those with first‐derivative processing in terms of classification accuracy and sample‐level accuracy, with PLSDA, XGBDA, and ANNDA achieving classification accuracies of 42.6%, 43.5%, and 40.0%, respectively, without first‐derivative processing. None of the models achieved accuracy above 70%, suggesting that the sample size was too small to adequately represent the entire dataset, indicating that training a model on data from a single event is insufficient. Interestingly, internal cross‐validation of these models yielded much higher accuracies, with most models reaching 98% or higher. This discrepancy suggests that machine learning models trained on small sample sizes, as in this case, should undergo external validation to provide a more accurate reflection of classification performance in real‐world applications.

**TABLE 4:** Various performances for machine learning models trained on Event 1 benchtop FTIR data tested with Event 2 benchtop FTIR data.

		External validation						Internal cross‐validation	
ML model	1st derivative?	Sample‐level accuracy	Accuracy	M sensitivity	F sensitivity	Global F1 score	MCC	Accuracy	MCC
PLSDA	No	43.5%	42.6%	78.0%	15.4%	42.6%	−0.085	100.0%	1.000
	Yes	17.4%	19.1%	30.0%	10.8%	19.1%	−0.610	100.0%	1.000
XGBDA	No	43.5%	43.5%	100.0%	0.0%	43.5%	–	98.0%	0.956
	Yes	39.1%	42.6%	24.0%	56.9%	42.6%	−0.199	100.0%	1.000
ANNDA	No	43.5%	40.0%	78.0%	10.8%	40.0%	−0.153	100.0%	1.000
	Yes	34.8%	30.4%	38.0%	24.6%	30.4%	−0.377	100.0%	1.000

Abbreviations: AML, machine learning; M, male; F, female; MCC, Matthew's correlation coefficient.

Additionally, we sought to determine if the Event 1 benchtop FTIR data, validated by handheld FTIR data, performed better than validation using Event 2 benchtop FTIR data. This hypothesis is based on the idea that both datasets use the same samples but different instruments. Compared with the model tested previously with Event 2 benchtop FTIR data, the model with Event 1 benchtop FTIR data performed better in all cases, regardless of whether first‐derivative filtering was applied (Table 5). Both PLSDA and ANNDA models showed improved performance after the first derivative was applied, achieving classification accuracies of 69.1% and 68.2%, respectively. However, XGBDA experienced a 12‐point decrease in classification accuracy, dropping from 58.2% to 46.4% with the addition of first‐derivative filtering. These results suggest that although both benchtop and handheld instruments can extract similar spectra from the same samples, models trained on small datasets tend to underperform, likely due to the limitations of the data volume and variability.

**TABLE 5:** Various performances for machine learning models trained on Event 1 benchtop FTIR data tested with Event 1 handheld FTIR data.

		External validation						Internal cross‐validation	
ML model	1st derivative?	Sample‐level accuracy	Accuracy	M sensitivity	F sensitivity	Global F1 score	MCC	Accuracy	MCC
PLSDA	No	50.0%	51.8%	74.3%	12.5%	51.8%	−0.156	100.0%	1.000
	Yes	68.2%	69.1%	71.4%	65.0%	69.1%	0.355	100.0%	1.000
XGBDA	No	59.1%	58.2%	91.0.4%	0.0%	58.1%	−0.182	98.0%	0.956
	Yes	45.5%	46.4%	35.7%	65.0%	46.4%	0.007	100.0%	1.000
ANNDA	No	45.5%	50.0%	74.3%	7.5%	50.0%	−0.223	100.0%	1.000
	Yes	68.2%	66.4%	71.4%	57.5%	66.4%	0.285	100.0%	1.000

Abbreviations: F, female; M, male; MCC, Matthew's correlation coefficient; ML, machine learning.

Finally, both benchtop datasets were combined to assess whether a larger, more robust dataset would improve model performance when tested with handheld FTIR data. We found that increasing the calibration dataset significantly enhanced model performance. For PLSDA and ANNDA, classification accuracies reached 90.0% and 94.5%, respectively, when first‐derivative filtering was applied (Table 6). Without the first‐derivative filter, the accuracies dropped to 63.6% and 68.2%, highlighting the importance of this preprocessing step. The sample‐level accuracy, which indicates how many samples' sexes were correctly predicted, was even higher, nearing 91% and 96% for PLSDA and ANNDA, respectively.

**TABLE 6:** Various performances for machine learning models trained on combined benchtop FTIR data tested with Event 1 handheld FTIR data.

		External validation						Internal cross‐validation	
ML model	1st derivative?	Sample‐level accuracy	Accuracy	M sensitivity	F sensitivity	Global F1 score	MCC	Accuracy	MCC
PLSDA	No	63.6%	63.6%	85.7%	25.0%	63.6%	0.134	97.7%	0.954
	Yes	90.9%	90.0%	90.0%	90.0%	90.0%	0.789	99.1%	0.981
XGBDA	No	36.4%	36.4%	0.0%	100.0%	36.4%	–	97.6%	0.953
	Yes	59.1%	58.2%	34.3%	100.0%	58.2%	0.399	99.5%	0.991
ANNDA	No	68.2%	68.2%	92.9%	25.0%	68.2%	0.250	99.5%	0.991
	Yes	95.5%	94.5%	92.9%	97.5%	94.5%	0.887	100.0%	1.000

Abbreviations: F, female; M, male; MCC, Matthews' correlation coefficient; ML, machine learning.

By contrast, XGBDA consistently underperformed, with classification accuracies of 58.2% and 36.4% with and without first‐derivative filtering. Internal cross‐validation performance showed a slight decrease compared to models trained only on Event 1 benchtop data, but this discrepancy brought the results closer to actual external validation performance, especially when first‐derivative filtering was used. These findings demonstrate that machine learning models can effectively differentiate male and female third instar *Ch. rufifacies* larvae from handheld FTIR data.

While our findings demonstrate that FTIR spectroscopy can distinguish male and female *Ch. rufifacies* larvae with high accuracy under controlled conditions, additional studies are needed to explore how ecological and environmental factors may influence the chemical signatures used for sex classification. In natural settings, *Ch. rufifacies* larvae develop within dense maggot masses that may consist of mixed‐sex clutches from multiple females. Under such conditions, factors like resource availability, larval crowding, and sex ratio could influence the expression of cuticular compounds, potentially altering the degree of sexual dimorphism observed in FTIR spectra. Increased competition or varying nutrient access could shift larval metabolic priorities, leading to plasticity in cuticular lipid, protein, or hydrocarbon profiles.

Moreover, temperature is a critical environmental variable that likely modulates cuticular chemistry. Since lipids and hydrocarbons play a role in desiccation resistance, sex‐specific FTIR signatures observed at one temperature (e.g., 25°C, as done here) may not persist at higher or lower rearing temperatures, where physiological adaptations may differ between sexes. The extent to which environmental stressors like heat or desiccation influence sexually dimorphic expression patterns remains an open question.

The potential for phenotypic plasticity in larval cuticular chemistry is supported by findings in other dipteran species. For instance, Berdan et al. (2019) showed that CHC expression in *Coelopa frigida* larvae varied with diet, while Thomas and Simmons (2011) demonstrated that social interactions (crowded vs. isolated) influenced CHC profiles in crickets [37, 38]. These results suggest that cuticular biochemistry is both dynamic and context‐dependent, emphasizing the need for future work to test the robustness of FTIR‐based sex classification under varied ecological conditions. Incorporating such factors into future model training will be essential for deploying these methods in realistic forensic or field‐based applications.

## 4 CONCLUSIONS

In summary, our analysis of male and female *Ch. rufifacies* third instar larvae using FTIR spectroscopy revealed significant differences in their spectral profiles, particularly in the fingerprint region, with males consistently exhibiting higher abundances of proteins, lipids, and hydrocarbons. Key peaks, such as 1053–1072, 1237, 1312–1314, 1340, 1396, 1450–1457, and 1539–1541 cm^−1^, were most notable in distinguishing the sexes, suggesting that the male larvae have a distinct cuticular composition and higher abundance of hydrocarbons, likely due to sexual selection. Machine learning models, including PLSDA and ANNDA, successfully classified male and female larvae, with performance notably improving when a larger calibration dataset was used, and first‐derivative filtering was applied. However, XGBDA consistently underperformed compared with PLSDA and ANNDA, with classification accuracies no higher than 58.2%, in contrast to its more optimistic cross‐validation results. This underperformance emphasizes the challenges of using small datasets for machine learning models and highlights the importance of external validation. These findings demonstrate that handheld and benchtop FTIR spectroscopy, coupled with high‐performing machine learning, can effectively differentiate between male and female third instar *Ch. rufifacies* larvae. However, to refine this approach for field application, future research should include time‐series analyses across larval development, such as sampling larvae every 12–24 h with multiple biological replicates, to determine how sex‐specific spectral differences evolve over time. Temperature and mixed‐brood conditions should also be systematically investigated to assess their potential effects on cuticular FTIR signatures. This developmental context will be crucial for validating models on field‐collected specimens that naturally vary in age, size, and growth rates. Continued efforts should also explore the potential of this method for differentiating sexes in earlier instars using the same nondestructive approach.

## FUNDING INFORMATION

This project was supported by Award No. 2020‐90663‐TX‐DU, awarded by the National Institute of Justice, Office of Justice Programs, U.S. Department of Justice.

## CONFLICT OF INTEREST STATEMENT

The authors have no conflicts of interest to declare.

## Supporting information


Data S1:

## Data Availability

The data that support the findings of this study are available from the corresponding author upon reasonable request.
