# Supplementary material for: Portable Fourier‐transform infrared spectroscopy and machine learning for sex determination in third instar Chrysomya rufifacies larvae
Source: J Forensic Sci. 2025 Apr 18;70(4):1468–79. doi: 10.1111/1556-4029.70054 (PMC12223342; doi:10.1111/1556-4029.70054)
Supplement: Supplementary file 1 — Data S1: [file JFO-70-1468-s001.docx]

**Supporting Information**


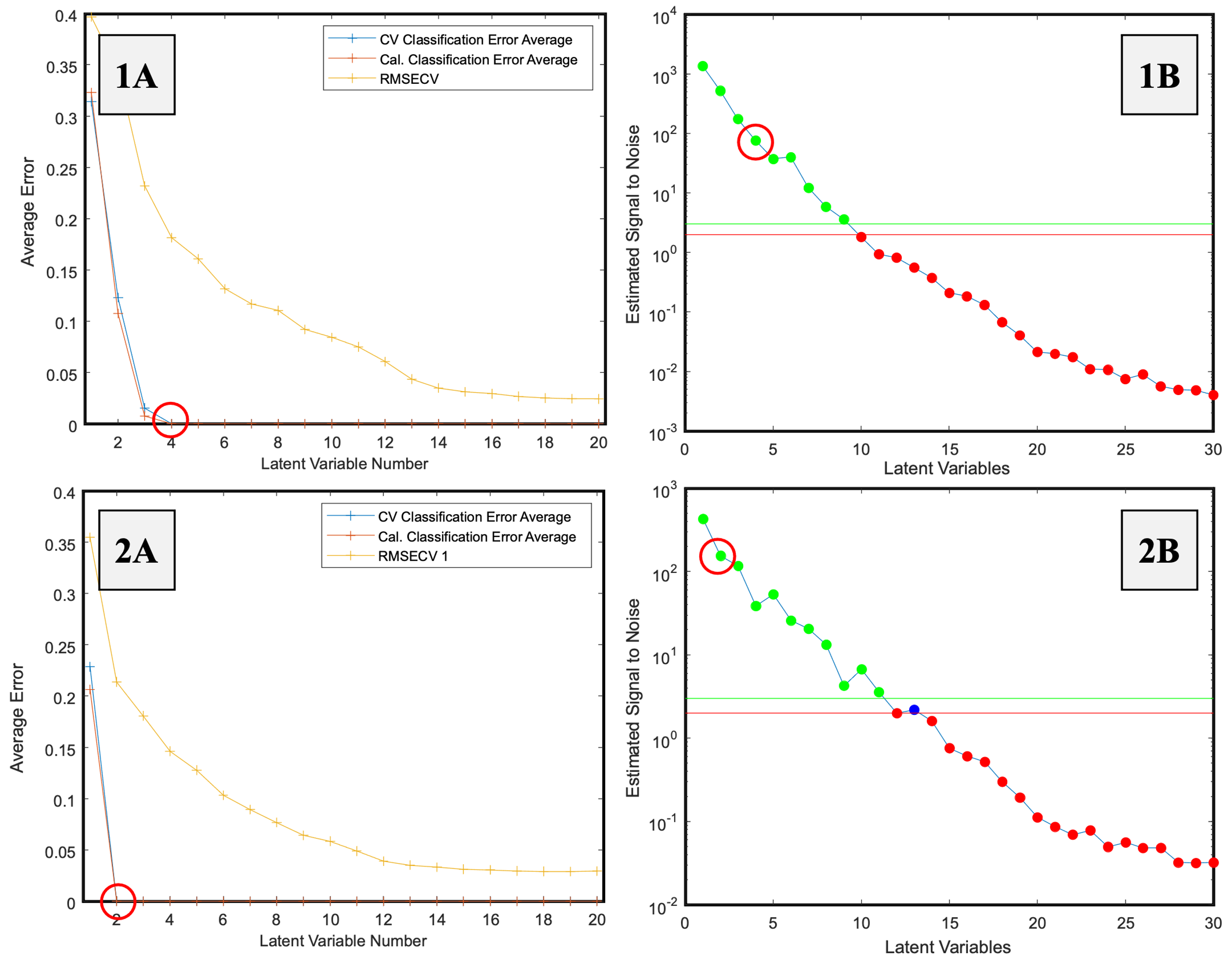


FIGURE S1 PLSDA optimization summary, specifically (A) the choice of model and (B) whether it included a variable with insufficient (estimated) SNR with (2) and without (1) 1^st^ derivative filtering for Event 1 benchtop FTIR-trained models.


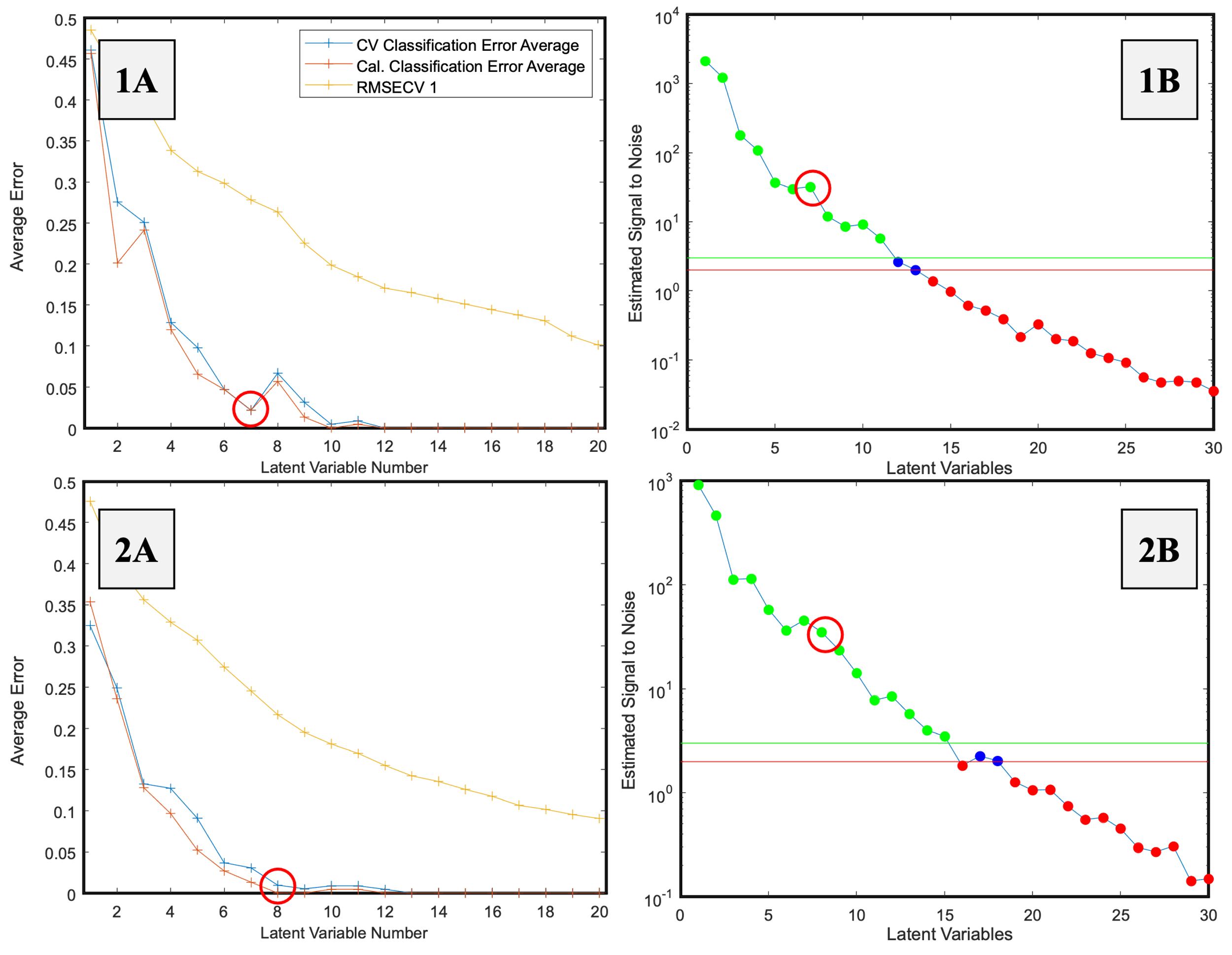


FIGURE S2 PLSDA optimization summary, specifically (A) the choice of model and (B) whether it included a variable with insufficient (estimated) SNR with (2) and without (1) 1^st^ derivative filtering for combined benchtop FTIR-trained models.


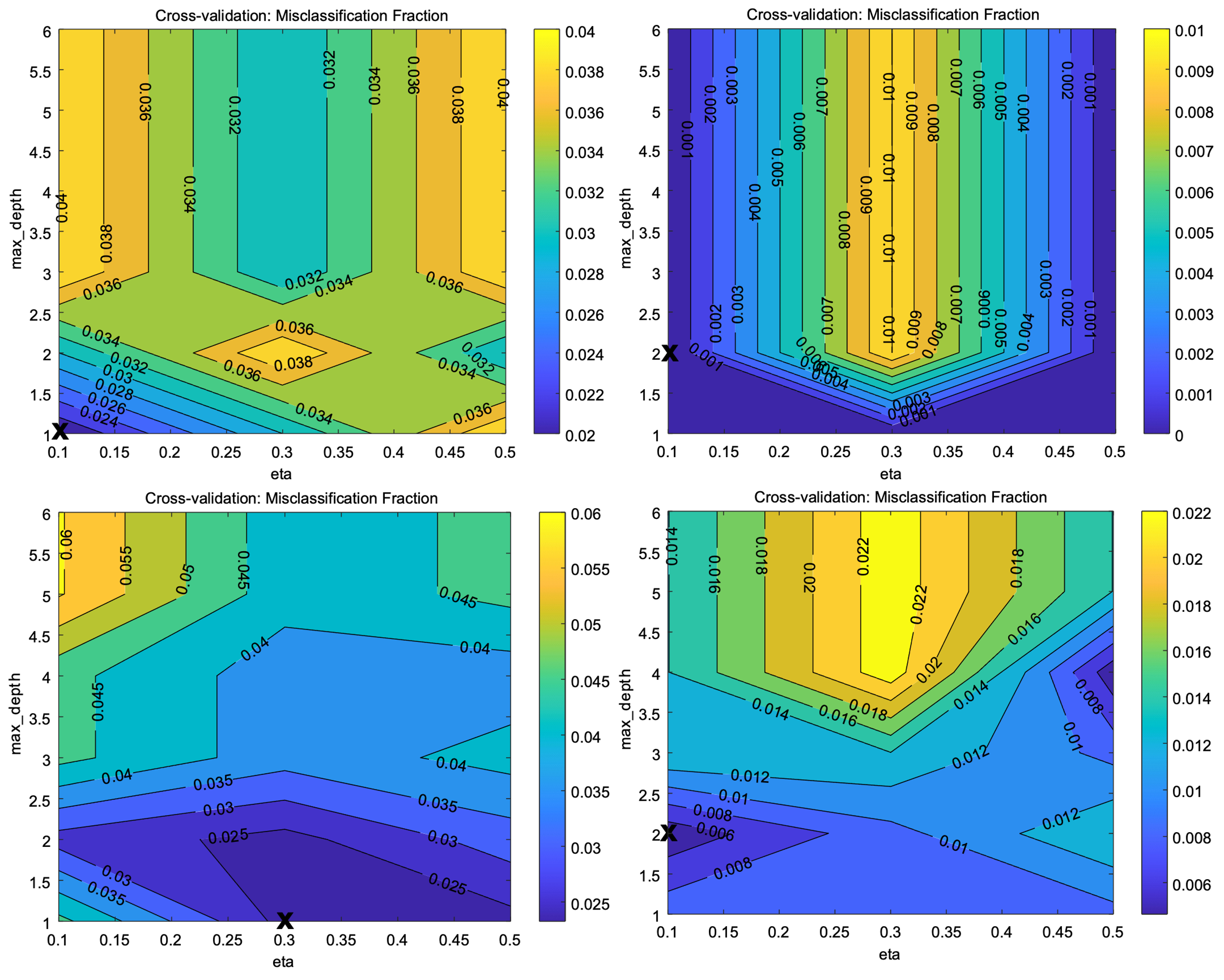


FIGRE S3 XGBDA optimization summary, specifically gradient map for model selection (X) based on optimal learning rate (eta) and number of decision trees (max_depth) during automatic hypertuning for with (B) and without (A) 1^st^ derivative filtering between models trained on (1) Event 1 benchtop FTIR data and (2) combined benchtop FTIR data.


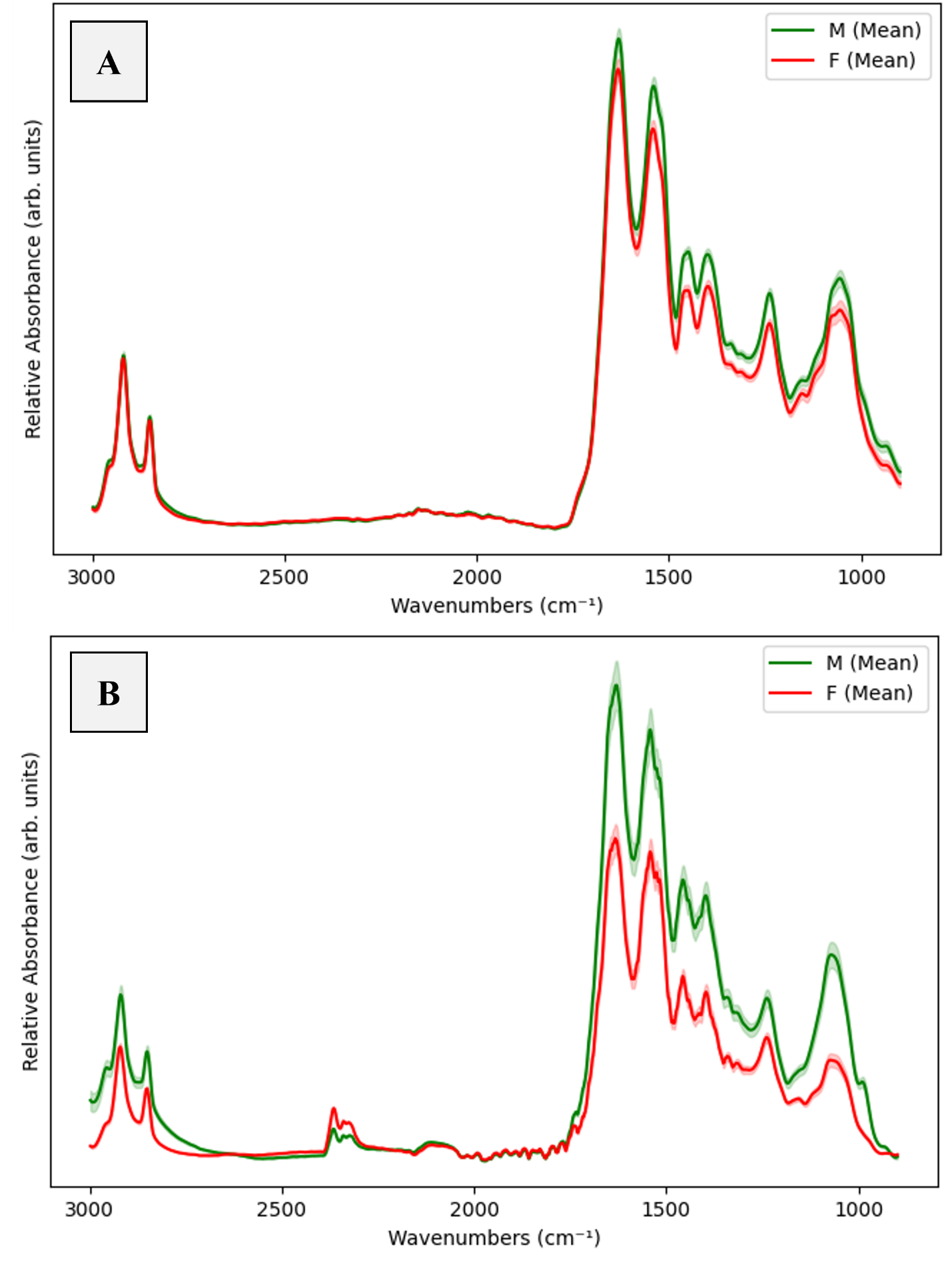


FIGURE S4 Mean (solid line) and standard error (filled regions) for (A) combined benchtop Events 1 and 2 and (B) handheld Event 1 datasets.


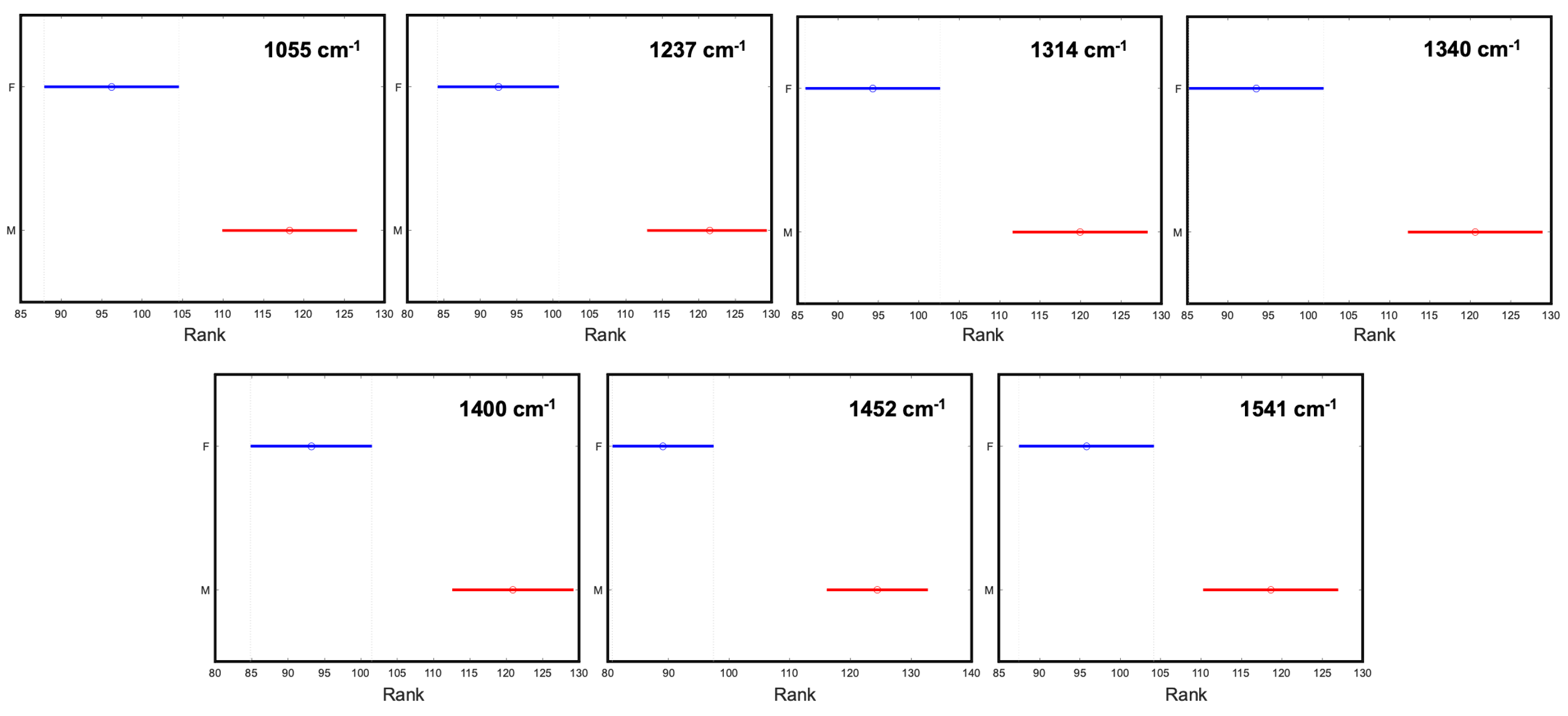


FIGURE S5 Pairwise comparison plots for sex-related differences throughout the Manhattan-plot-overlapped, combined benchtop FTIR data.


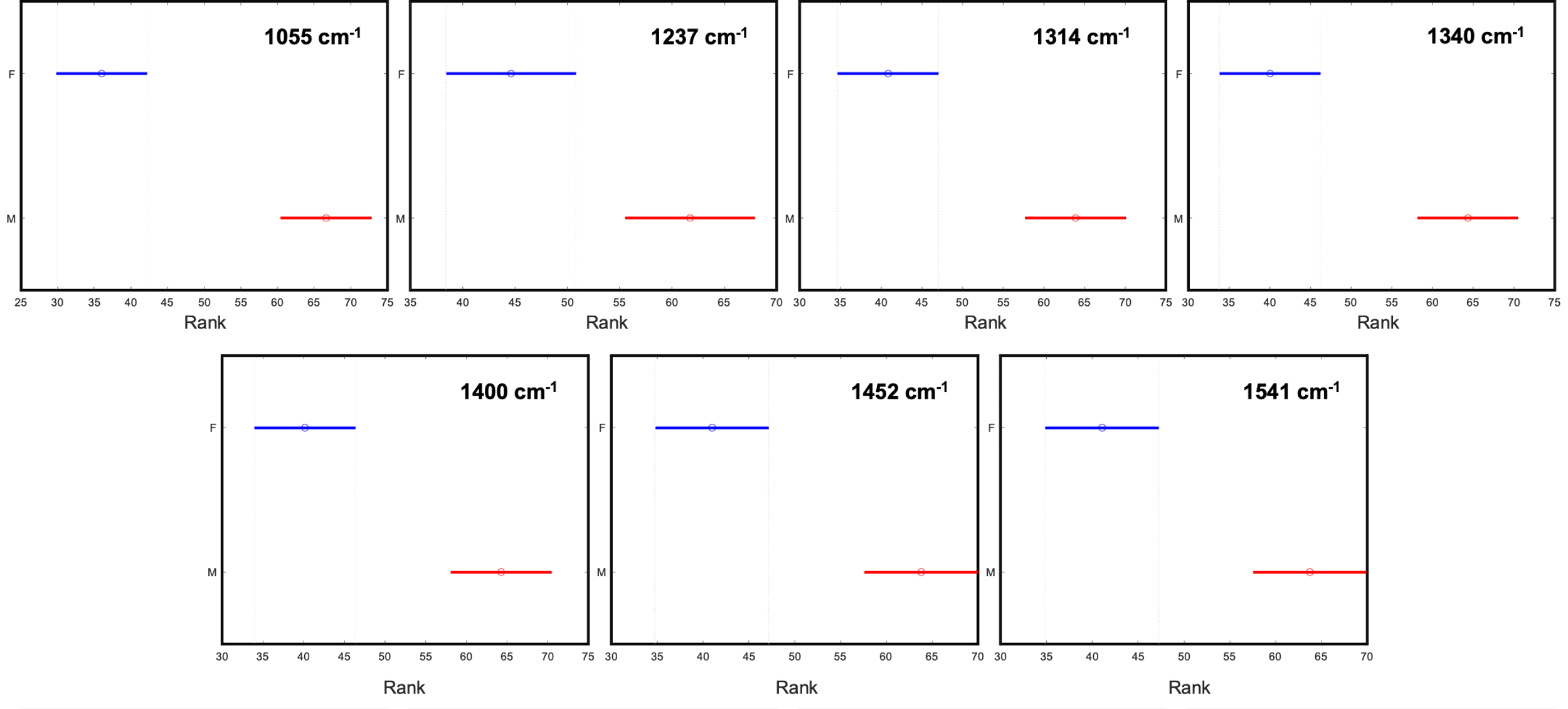


FIGURE S6 Pairwise comparison plots for sex-related differences throughout the Manhattan-plot-overlapped, handheld FTIR data.

TABLE S1 Event 1 benchtop-trained PLSDA model with mean centering-only validated on Event 2 benchtop FTIR data.

| **Label** | **Actual Sex** | **Predicted as F** | **Predicted as M** | **% Correct** | **ID Correct (Y/N)?** | **Label** | **Actual Sex** | **Predicted as F** | **Predicted as M** | **% Correct** | **ID Correct (Y/N)?** |
| --- | --- | --- | --- | --- | --- | --- | --- | --- | --- | --- | --- |
| BT25 | M | 0 | 5 | 100 | Y | BT39 | M | 1 | 4 | 80 | Y |
| BT26 | M | 0 | 5 | 100 | Y | BT40 | F | 0 | 5 | 0 | N |
| BT27 | M | 0 | 5 | 100 | Y | BT41 | F | 0 | 5 | 0 | N |
| BT28 | M | 0 | 5 | 100 | Y | BT42 | F | 5 | 0 | 100 | Y |
| BT29 | M | 0 | 5 | 100 | Y | BT43 | F | 0 | 5 | 0 | N |
| BT30 | F | 0 | 5 | 0 | N | BT44 | F | 0 | 5 | 0 | N |
| BT31 | F | 0 | 5 | 0 | N | BT45 | F | 0 | 5 | 0 | N |
| BT34 | F | 0 | 5 | 0 | N | BT46 | F | 0 | 5 | 0 | N |
| BT35 | M | 0 | 5 | 100 | Y | BT47 | F | 0 | 5 | 0 | N |
| BT36 | M | 5 | 0 | 0 | N | BT48 | F | 5 | 0 | 100 | Y |
| BT37 | M | 0 | 5 | 100 | Y | BT49 | F | 0 | 5 | 0 | N |
| BT38 | M | 5 | 0 | 0 | N | -- | -- | -- | -- | -- | -- |

TABLE S2 Event 1 benchtop-trained PLSDA model with 1^st^ derivative and mean centering processing validated on Event 2 benchtop FTIR data.

| **Label** | **Actual Sex** | **Predicted as F** | **Predicted as M** | **% Correct** | **ID Correct (Y/N)?** | **Label** | **Actual Sex** | **Predicted as F** | **Predicted as M** | **% Correct** | **ID Correct (Y/N)?** |
| --- | --- | --- | --- | --- | --- | --- | --- | --- | --- | --- | --- |
| BT25 | M | 5 | 0 | 0 | N | BT39 | M | 1 | 4 | 80 | Y |
| BT26 | M | 5 | 0 | 0 | N | BT40 | F | 0 | 5 | 0 | N |
| BT27 | M | 4 | 1 | 20 | N | BT41 | F | 0 | 5 | 0 | N |
| BT28 | M | 5 | 0 | 0 | N | BT42 | F | 2 | 3 | 40 | N |
| BT29 | M | 5 | 0 | 0 | N | BT43 | F | 0 | 5 | 0 | N |
| BT30 | F | 0 | 5 | 0 | N | BT44 | F | 0 | 5 | 0 | N |
| BT31 | F | 0 | 5 | 0 | N | BT45 | F | 0 | 5 | 0 | N |
| BT34 | F | 0 | 5 | 0 | N | BT46 | F | 0 | 5 | 0 | N |
| BT35 | M | 0 | 5 | 100 | Y | BT47 | F | 0 | 5 | 0 | N |
| BT36 | M | 5 | 0 | 0 | N | BT48 | F | 1 | 4 | 20 | N |
| BT37 | M | 0 | 5 | 100 | Y | BT49 | F | 4 | 1 | 80 | Y |
| BT38 | M | 5 | 0 | 0 | N | -- | -- | -- | -- | -- | -- |

TABLE S3 Event 1 benchtop-trained XGBDA model with mean centering-only validated on Event 2 benchtop FTIR data.

| **Label** | **Actual Sex** | **Predicted as F** | **Predicted as M** | **% Correct** | **ID Correct (Y/N)?** | **Label** | **Actual Sex** | **Predicted as F** | **Predicted as M** | **% Correct** | **ID Correct (Y/N)?** |
| --- | --- | --- | --- | --- | --- | --- | --- | --- | --- | --- | --- |
| BT25 | M | 0 | 5 | 100 | Y | BT39 | M | 0 | 5 | 100 | Y |
| BT26 | M | 0 | 5 | 100 | Y | BT40 | F | 0 | 5 | 0 | N |
| BT27 | M | 0 | 5 | 100 | Y | BT41 | F | 0 | 5 | 0 | N |
| BT28 | M | 0 | 5 | 100 | Y | BT42 | F | 0 | 5 | 0 | N |
| BT29 | M | 0 | 5 | 100 | Y | BT43 | F | 0 | 5 | 0 | N |
| BT30 | F | 0 | 5 | 0 | N | BT44 | F | 0 | 5 | 0 | N |
| BT31 | F | 0 | 5 | 0 | N | BT45 | F | 0 | 5 | 0 | N |
| BT34 | F | 0 | 5 | 0 | N | BT46 | F | 0 | 5 | 0 | N |
| BT35 | M | 0 | 5 | 100 | Y | BT47 | F | 0 | 5 | 0 | N |
| BT36 | M | 0 | 5 | 100 | Y | BT48 | F | 0 | 5 | 0 | N |
| BT37 | M | 0 | 5 | 100 | Y | BT49 | F | 0 | 5 | 0 | N |
| BT38 | M | 0 | 5 | 100 | Y | -- | -- | -- | -- | -- | -- |

TABLE S4 Event 1 benchtop-trained XGBDA model with 1^st^ derivative and mean centering processing validated on Event 2 benchtop FTIR data.

| **Label** | **Actual Sex** | **Predicted as F** | **Predicted as M** | **% Correct** | **ID Correct (Y/N)?** | **Label** | **Actual Sex** | **Predicted as F** | **Predicted as M** | **% Correct** | **ID Correct (Y/N)?** |
| --- | --- | --- | --- | --- | --- | --- | --- | --- | --- | --- | --- |
| BT25 | M | 5 | 0 | 0 | N | BT39 | M | 1 | 4 | 80 | Y |
| BT26 | M | 5 | 0 | 0 | N | BT40 | F | 2 | 3 | 40 | N |
| BT27 | M | 5 | 0 | 0 | N | BT41 | F | 5 | 0 | 100 | Y |
| BT28 | M | 5 | 0 | 0 | N | BT42 | F | 5 | 0 | 100 | Y |
| BT29 | M | 5 | 0 | 0 | N | BT43 | F | 2 | 3 | 40 | N |
| BT30 | F | 3 | 2 | 60 | Y | BT44 | F | 4 | 1 | 80 | Y |
| BT31 | F | 2 | 3 | 40 | N | BT45 | F | 0 | 5 | 0 | N |
| BT34 | F | 0 | 5 | 0 | N | BT46 | F | 0 | 5 | 0 | N |
| BT35 | M | 0 | 5 | 100 | Y | BT47 | F | 4 | 1 | 80 | Y |
| BT36 | M | 3 | 2 | 40 | N | BT48 | F | 5 | 0 | 100 | Y |
| BT37 | M | 4 | 1 | 20 | N | BT49 | F | 5 | 0 | 100 | Y |
| BT38 | M | 5 | 0 | 0 | N | -- | -- | -- | -- | -- | -- |

TABLE S5 Event 1 benchtop-trained ANNDA model with mean centering-only validated on Event 2 benchtop FTIR data.

| **Label** | **Actual Sex** | **Predicted as F** | **Predicted as M** | **% Correct** | **ID Correct (Y/N)?** | **Label** | **Actual Sex** | **Predicted as F** | **Predicted as M** | **% Correct** | **ID Correct (Y/N)?** |
| --- | --- | --- | --- | --- | --- | --- | --- | --- | --- | --- | --- |
| BT25 | M | 0 | 5 | 100 | Y | BT39 | M | 1 | 4 | 80 | Y |
| BT26 | M | 0 | 5 | 100 | Y | BT40 | F | 0 | 5 | 0 | N |
| BT27 | M | 0 | 5 | 100 | Y | BT41 | F | 0 | 5 | 0 | N |
| BT28 | M | 0 | 5 | 100 | Y | BT42 | F | 3 | 2 | 60 | Y |
| BT29 | M | 0 | 5 | 100 | Y | BT43 | F | 0 | 5 | 0 | N |
| BT30 | F | 0 | 5 | 0 | N | BT44 | F | 0 | 5 | 0 | N |
| BT31 | F | 0 | 5 | 0 | N | BT45 | F | 0 | 5 | 0 | N |
| BT34 | F | 0 | 5 | 0 | N | BT46 | F | 0 | 5 | 0 | N |
| BT35 | M | 0 | 5 | 100 | Y | BT47 | F | 0 | 5 | 0 | N |
| BT36 | M | 5 | 0 | 0 | N | BT48 | F | 4 | 1 | 80 | Y |
| BT37 | M | 0 | 5 | 100 | Y | BT49 | F | 0 | 5 | 0 | N |
| BT38 | M | 5 | 0 | 0 | N | -- | -- | -- | -- | -- | -- |

TABLE S6 Event 1 benchtop-trained ANNDA model with 1^st^ derivative and mean centering processing validated on Event 2 benchtop FTIR data.

| **Label** | **Actual Sex** | **Predicted as F** | **Predicted as M** | **% Correct** | **ID Correct (Y/N)?** | **Label** | **Actual Sex** | **Predicted as F** | **Predicted as M** | **% Correct** | **ID Correct (Y/N)?** |
| --- | --- | --- | --- | --- | --- | --- | --- | --- | --- | --- | --- |
| BT25 | M | 5 | 0 | 0 | N | BT39 | M | 1 | 4 | 80 | Y |
| BT26 | M | 5 | 0 | 0 | N | BT40 | F | 5 | 0 | 100 | Y |
| BT27 | M | 0 | 5 | 100 | Y | BT41 | F | 0 | 5 | 0 | N |
| BT28 | M | 5 | 0 | 0 | N | BT42 | F | 3 | 2 | 60 | Y |
| BT29 | M | 5 | 0 | 0 | N | BT43 | F | 0 | 5 | 0 | N |
| BT30 | F | 0 | 5 | 0 | N | BT44 | F | 0 | 5 | 0 | N |
| BT31 | F | 0 | 5 | 0 | N | BT45 | F | 0 | 5 | 0 | N |
| BT34 | F | 0 | 5 | 0 | N | BT46 | F | 0 | 5 | 0 | N |
| BT35 | M | 0 | 5 | 100 | Y | BT47 | F | 0 | 5 | 0 | N |
| BT36 | M | 5 | 0 | 0 | N | BT48 | F | 3 | 2 | 60 | Y |
| BT37 | M | 0 | 5 | 100 | Y | BT49 | F | 5 | 0 | 100 | Y |
| BT38 | M | 5 | 0 | 0 | N | -- | -- | -- | -- | -- | -- |

TABLE S7 Event 1 benchtop-trained PLSDA model with mean centering-only validated on Event 1 handheld FTIR data.

| **Label** | **Actual Sex** | **Predicted as F** | **Predicted as M** | **% Correct** | **ID Correct (Y/N)?** | **Label** | **Actual Sex** | **Predicted as F** | **Predicted as M** | **% Correct** | **ID Correct (Y/N)?** |
| --- | --- | --- | --- | --- | --- | --- | --- | --- | --- | --- | --- |
| HH01 | M | 5 | 0 | 0 | N | HH12 | M | 0 | 5 | 100 | Y |
| HH02 | M | 0 | 5 | 100 | Y | HH13 | M | 0 | 5 | 100 | Y |
| HH03 | M | 0 | 5 | 100 | Y | HH14 | M | 0 | 5 | 100 | Y |
| HH04 | M | 0 | 5 | 100 | Y | HH15 | F | 0 | 5 | 0 | N |
| HH05 | M | 0 | 5 | 100 | Y | HH16 | F | 0 | 5 | 0 | N |
| HH06 | M | 5 | 0 | 0 | N | HH17 | F | 0 | 5 | 0 | N |
| HH07 | M | 5 | 0 | 0 | N | HH18 | F | 0 | 5 | 0 | N |
| HH08 | M | 0 | 5 | 100 | Y | HH20 | F | 0 | 5 | 0 | N |
| HH09 | M | 3 | 2 | 40 | N | HH21 | F | 0 | 5 | 0 | N |
| HH10 | M | 0 | 5 | 100 | Y | HH22 | F | 0 | 5 | 0 | N |
| HH11 | M | 0 | 5 | 100 | Y | HH24 | F | 5 | 0 | 100 | Y |

TABLE S8 Event 1 benchtop-trained PLSDA model with 1^st^ derivative and mean centering processing validated on Event 1 handheld FTIR data.

| **Label** | **Actual Sex** | **Predicted as F** | **Predicted as M** | **% Correct** | **ID Correct (Y/N)?** | **Label** | **Actual Sex** | **Predicted as F** | **Predicted as M** | **% Correct** | **ID Correct (Y/N)?** |
| --- | --- | --- | --- | --- | --- | --- | --- | --- | --- | --- | --- |
| HH01 | M | 5 | 0 | 0 | N | HH12 | M | 0 | 5 | 100 | Y |
| HH02 | M | 0 | 5 | 100 | Y | HH13 | M | 0 | 5 | 100 | Y |
| HH03 | M | 0 | 5 | 100 | Y | HH14 | M | 0 | 5 | 100 | Y |
| HH04 | M | 0 | 5 | 100 | Y | HH15 | F | 0 | 5 | 0 | N |
| HH05 | M | 0 | 5 | 100 | Y | HH16 | F | 3 | 2 | 60 | Y |
| HH06 | M | 5 | 0 | 0 | N | HH17 | F | 5 | 0 | 100 | Y |
| HH07 | M | 5 | 0 | 0 | N | HH18 | F | 5 | 0 | 100 | Y |
| HH08 | M | 0 | 5 | 100 | Y | HH20 | F | 5 | 0 | 100 | Y |
| HH09 | M | 5 | 0 | 0 | N | HH21 | F | 0 | 5 | 0 | N |
| HH10 | M | 0 | 5 | 100 | Y | HH22 | F | 0 | 5 | 0 | N |
| HH11 | M | 0 | 5 | 100 | Y | HH24 | F | 5 | 0 | 100 | Y |

TABLE S9 Event 1 benchtop-trained XGBDA model with mean centering-only validated on Event 1 handheld FTIR data.

| **Label** | **Actual Sex** | **Predicted as F** | **Predicted as M** | **% Correct** | **ID Correct (Y/N)?** | **Label** | **Actual Sex** | **Predicted as F** | **Predicted as M** | **% Correct** | **ID Correct (Y/N)?** |
| --- | --- | --- | --- | --- | --- | --- | --- | --- | --- | --- | --- |
| HH01 | M | 0 | 5 | 100 | Y | HH12 | M | 0 | 5 | 100 | Y |
| HH02 | M | 0 | 5 | 100 | Y | HH13 | M | 0 | 5 | 100 | Y |
| HH03 | M | 0 | 5 | 100 | Y | HH14 | M | 0 | 5 | 100 | Y |
| HH04 | M | 0 | 5 | 100 | Y | HH15 | F | 0 | 5 | 0 | N |
| HH05 | M | 0 | 5 | 100 | Y | HH16 | F | 0 | 5 | 0 | N |
| HH06 | M | 1 | 4 | 80 | Y | HH17 | F | 0 | 5 | 0 | N |
| HH07 | M | 5 | 0 | 0 | N | HH18 | F | 0 | 5 | 0 | N |
| HH08 | M | 0 | 5 | 100 | Y | HH20 | F | 0 | 5 | 0 | N |
| HH09 | M | 0 | 5 | 100 | Y | HH21 | F | 0 | 5 | 0 | N |
| HH10 | M | 0 | 5 | 100 | Y | HH22 | F | 0 | 5 | 0 | N |
| HH11 | M | 0 | 5 | 100 | Y | HH24 | F | 0 | 5 | 0 | N |

TABLE S10 Event 1 benchtop-trained XGBDA model with 1^st^ derivative and mean centering processing validated on Event 1 handheld FTIR data.

| **Label** | **Actual Sex** | **Predicted as F** | **Predicted as M** | **% Correct** | **ID Correct (Y/N)?** | **Label** | **Actual Sex** | **Predicted as F** | **Predicted as M** | **% Correct** | **ID Correct (Y/N)?** |
| --- | --- | --- | --- | --- | --- | --- | --- | --- | --- | --- | --- |
| HH01 | M | 5 | 0 | 0 | N | HH12 | M | 5 | 0 | 0 | N |
| HH02 | M | 0 | 5 | 100 | Y | HH13 | M | 5 | 0 | 0 | N |
| HH03 | M | 0 | 5 | 100 | Y | HH14 | M | 0 | 5 | 100 | Y |
| HH04 | M | 0 | 5 | 100 | Y | HH15 | F | 0 | 5 | 0 | N |
| HH05 | M | 0 | 5 | 100 | Y | HH16 | F | 5 | 0 | 100 | Y |
| HH06 | M | 5 | 0 | 0 | N | HH17 | F | 5 | 0 | 100 | Y |
| HH07 | M | 5 | 0 | 0 | N | HH18 | F | 5 | 0 | 100 | Y |
| HH08 | M | 5 | 0 | 0 | N | HH20 | F | 5 | 0 | 100 | Y |
| HH09 | M | 5 | 0 | 0 | N | HH21 | F | 0 | 5 | 0 | N |
| HH10 | M | 5 | 0 | 0 | N | HH22 | F | 1 | 4 | 20 | N |
| HH11 | M | 5 | 0 | 0 | N | HH24 | F | 5 | 0 | 100 | Y |

TABLE S11 Event 1 benchtop-trained ANNDA model with mean centering-only validated on Event 1 handheld FTIR data.

| **Label** | **Actual Sex** | **Predicted as F** | **Predicted as M** | **% Correct** | **ID Correct (Y/N)?** | **Label** | **Actual Sex** | **Predicted as F** | **Predicted as M** | **% Correct** | **ID Correct (Y/N)?** |
| --- | --- | --- | --- | --- | --- | --- | --- | --- | --- | --- | --- |
| HH01 | M | 5 | 0 | 0 | N | HH12 | M | 0 | 5 | 100 | Y |
| HH02 | M | 0 | 5 | 100 | Y | HH13 | M | 0 | 5 | 100 | Y |
| HH03 | M | 0 | 5 | 100 | Y | HH14 | M | 0 | 5 | 100 | Y |
| HH04 | M | 0 | 5 | 100 | Y | HH15 | F | 0 | 5 | 0 | N |
| HH05 | M | 0 | 5 | 100 | Y | HH16 | F | 0 | 5 | 0 | N |
| HH06 | M | 5 | 0 | 0 | N | HH17 | F | 0 | 5 | 0 | N |
| HH07 | M | 5 | 0 | 0 | N | HH18 | F | 0 | 5 | 0 | N |
| HH08 | M | 0 | 5 | 100 | Y | HH20 | F | 0 | 5 | 0 | N |
| HH09 | M | 3 | 2 | 40 | N | HH21 | F | 0 | 5 | 0 | N |
| HH10 | M | 0 | 5 | 100 | Y | HH22 | F | 0 | 5 | 0 | N |
| HH11 | M | 0 | 5 | 100 | Y | HH24 | F | 3 | 2 | 60 | Y |

TABLE S12 Event 1 benchtop-trained ANNDA model with 1^st^ derivative and mean centering processing validated on Event 1 handheld FTIR data.

| **Label** | **Actual Sex** | **Predicted as F** | **Predicted as M** | **% Correct** | **ID Correct (Y/N)?** | **Label** | **Actual Sex** | **Predicted as F** | **Predicted as M** | **% Correct** | **ID Correct (Y/N)?** |
| --- | --- | --- | --- | --- | --- | --- | --- | --- | --- | --- | --- |
| HH01 | M | 5 | 0 | 0 | N | HH12 | M | 0 | 5 | 100 | Y |
| HH02 | M | 0 | 5 | 100 | Y | HH13 | M | 0 | 5 | 100 | Y |
| HH03 | M | 0 | 5 | 100 | Y | HH14 | M | 0 | 5 | 100 | Y |
| HH04 | M | 0 | 5 | 100 | Y | HH15 | F | 0 | 5 | 0 | N |
| HH05 | M | 0 | 5 | 100 | Y | HH16 | F | 0 | 5 | 0 | N |
| HH06 | M | 5 | 0 | 0 | N | HH17 | F | 0 | 5 | 0 | N |
| HH07 | M | 5 | 0 | 0 | N | HH18 | F | 5 | 0 | 100 | Y |
| HH08 | M | 0 | 5 | 100 | Y | HH20 | F | 5 | 0 | 100 | Y |
| HH09 | M | 5 | 0 | 0 | N | HH21 | F | 5 | 0 | 100 | Y |
| HH10 | M | 0 | 5 | 100 | Y | HH22 | F | 5 | 0 | 100 | Y |
| HH11 | M | 0 | 5 | 100 | Y | HH24 | F | 5 | 0 | 100 | Y |

TABLE S13 Combined benchtop-trained PLSDA model with mean centering-only validated on Event 1 handheld FTIR data.

| **Label** | **Actual Sex** | **Predicted as F** | **Predicted as M** | **% Correct** | **ID Correct (Y/N)?** | **Label** | **Actual Sex** | **Predicted as F** | **Predicted as M** | **% Correct** | **ID Correct (Y/N)?** |
| --- | --- | --- | --- | --- | --- | --- | --- | --- | --- | --- | --- |
| HH01 | M | 5 | 0 | 0 | N | HH12 | M | 0 | 5 | 100 | Y |
| HH02 | M | 0 | 5 | 100 | Y | HH13 | M | 0 | 5 | 100 | Y |
| HH03 | M | 0 | 5 | 100 | Y | HH14 | M | 0 | 5 | 100 | Y |
| HH04 | M | 0 | 5 | 100 | Y | HH15 | F | 0 | 5 | 0 | N |
| HH05 | M | 0 | 5 | 100 | Y | HH16 | F | 0 | 5 | 0 | N |
| HH06 | M | 0 | 5 | 100 | Y | HH17 | F | 0 | 5 | 0 | N |
| HH07 | M | 5 | 0 | 0 | N | HH18 | F | 0 | 5 | 0 | N |
| HH08 | M | 0 | 5 | 100 | Y | HH20 | F | 5 | 0 | 100 | Y |
| HH09 | M | 0 | 5 | 100 | Y | HH21 | F | 0 | 5 | 0 | N |
| HH10 | M | 0 | 5 | 100 | Y | HH22 | F | 0 | 5 | 0 | N |
| HH11 | M | 0 | 5 | 100 | Y | HH24 | F | 5 | 0 | 100 | Y |

TABLE S14 Combined benchtop-trained PLSDA model with 1^st^ derivative and mean centering processing validated on Event 1 handheld FTIR data.

| **Label** | **Actual Sex** | **Predicted as F** | **Predicted as M** | **% Correct** | **ID Correct (Y/N)?** | **Label** | **Actual Sex** | **Predicted as F** | **Predicted as M** | **% Correct** | **ID Correct (Y/N)?** |
| --- | --- | --- | --- | --- | --- | --- | --- | --- | --- | --- | --- |
| HH01 | M | 5 | 0 | 0 | N | HH12 | M | 0 | 5 | 100 | Y |
| HH02 | M | 0 | 5 | 100 | Y | HH13 | M | 0 | 5 | 100 | Y |
| HH03 | M | 0 | 5 | 100 | Y | HH14 | M | 0 | 5 | 100 | Y |
| HH04 | M | 0 | 5 | 100 | Y | HH15 | F | 1 | 4 | 20 | N |
| HH05 | M | 0 | 5 | 100 | Y | HH16 | F | 5 | 0 | 100 | Y |
| HH06 | M | 1 | 4 | 80 | Y | HH17 | F | 5 | 0 | 100 | Y |
| HH07 | M | 0 | 5 | 100 | Y | HH18 | F | 5 | 0 | 100 | Y |
| HH08 | M | 0 | 5 | 100 | Y | HH20 | F | 5 | 0 | 100 | Y |
| HH09 | M | 1 | 4 | 80 | Y | HH21 | F | 5 | 0 | 100 | Y |
| HH10 | M | 0 | 5 | 100 | Y | HH22 | F | 5 | 0 | 100 | Y |
| HH11 | M | 0 | 5 | 100 | Y | HH24 | F | 5 | 0 | 100 | Y |

TABLE S15 Combined benchtop-trained XGBDA model with mean centering-only validated on Event 1 handheld FTIR data.

| **Label** | **Actual Sex** | **Predicted as F** | **Predicted as M** | **% Correct** | **ID Correct (Y/N)?** | **Label** | **Actual Sex** | **Predicted as F** | **Predicted as M** | **% Correct** | **ID Correct (Y/N)?** |
| --- | --- | --- | --- | --- | --- | --- | --- | --- | --- | --- | --- |
| HH01 | M | 5 | 0 | 0 | N | HH12 | M | 5 | 0 | 0 | N |
| HH02 | M | 5 | 0 | 0 | N | HH13 | M | 5 | 0 | 0 | N |
| HH03 | M | 5 | 0 | 0 | N | HH14 | M | 5 | 0 | 0 | N |
| HH04 | M | 5 | 0 | 0 | N | HH15 | F | 0 | 5 | 100 | Y |
| HH05 | M | 5 | 0 | 0 | N | HH16 | F | 0 | 5 | 100 | Y |
| HH06 | M | 5 | 0 | 0 | N | HH17 | F | 0 | 5 | 100 | Y |
| HH07 | M | 5 | 0 | 0 | N | HH18 | F | 0 | 5 | 100 | Y |
| HH08 | M | 5 | 0 | 0 | N | HH20 | F | 0 | 5 | 100 | Y |
| HH09 | M | 5 | 0 | 0 | N | HH21 | F | 0 | 5 | 100 | Y |
| HH10 | M | 5 | 0 | 0 | N | HH22 | F | 0 | 5 | 100 | Y |
| HH11 | M | 5 | 0 | 0 | N | HH24 | F | 0 | 5 | 100 | Y |

TABLE S16 Combined benchtop-trained XGBDA model with 1^st^ derivative and mean centering processing validated on Event 1 handheld FTIR data.

| **Label** | **Actual Sex** | **Predicted as F** | **Predicted as M** | **% Correct** | **ID Correct (Y/N)?** | **Label** | **Actual Sex** | **Predicted as F** | **Predicted as M** | **% Correct** | **ID Correct (Y/N)?** |
| --- | --- | --- | --- | --- | --- | --- | --- | --- | --- | --- | --- |
| HH01 | M | 2 | 3 | 60 | Y | HH12 | M | 5 | 0 | 0 | N |
| HH02 | M | 5 | 0 | 0 | N | HH13 | M | 2 | 3 | 60 | Y |
| HH03 | M | 5 | 0 | 0 | N | HH14 | M | 0 | 5 | 100 | Y |
| HH04 | M | 5 | 0 | 0 | N | HH15 | F | 5 | 0 | 100 | Y |
| HH05 | M | 5 | 0 | 0 | N | HH16 | F | 5 | 0 | 100 | Y |
| HH06 | M | 0 | 5 | 100 | Y | HH17 | F | 5 | 0 | 100 | Y |
| HH07 | M | 5 | 0 | 0 | N | HH18 | F | 5 | 0 | 100 | Y |
| HH08 | M | 5 | 0 | 0 | N | HH20 | F | 5 | 0 | 100 | Y |
| HH09 | M | 3 | 2 | 40 | N | HH21 | F | 5 | 0 | 100 | Y |
| HH10 | M | 4 | 1 | 20 | N | HH22 | F | 5 | 0 | 100 | Y |
| HH11 | M | 0 | 5 | 100 | Y | HH24 | F | 5 | 0 | 100 | Y |

TABLE S17 Combined benchtop-trained ANNDA model with mean centering-only validated on Event 1 handheld FTIR data.

| **Label** | **Actual Sex** | **Predicted as F** | **Predicted as M** | **% Correct** | **ID Correct (Y/N)?** | **Label** | **Actual Sex** | **Predicted as F** | **Predicted as M** | **% Correct** | **ID Correct (Y/N)?** |
| --- | --- | --- | --- | --- | --- | --- | --- | --- | --- | --- | --- |
| HH01 | M | 5 | 0 | 0 | N | HH12 | M | 0 | 5 | 100 | Y |
| HH02 | M | 0 | 5 | 100 | Y | HH13 | M | 0 | 5 | 100 | Y |
| HH03 | M | 0 | 5 | 100 | Y | HH14 | M | 0 | 5 | 100 | Y |
| HH04 | M | 0 | 5 | 100 | Y | HH15 | F | 0 | 5 | 0 | N |
| HH05 | M | 0 | 5 | 100 | Y | HH16 | F | 0 | 5 | 0 | N |
| HH06 | M | 0 | 5 | 100 | Y | HH17 | F | 0 | 5 | 0 | N |
| HH07 | M | 0 | 5 | 100 | Y | HH18 | F | 0 | 5 | 0 | N |
| HH08 | M | 0 | 5 | 100 | Y | HH20 | F | 5 | 0 | 100 | Y |
| HH09 | M | 0 | 5 | 100 | Y | HH21 | F | 0 | 5 | 0 | N |
| HH10 | M | 0 | 5 | 100 | Y | HH22 | F | 0 | 5 | 0 | N |
| HH11 | M | 0 | 5 | 100 | Y | HH24 | F | 5 | 0 | 100 | Y |

TABLE S18 Combined benchtop-trained ANNDA model with 1^st^ derivative and mean centering processing validated on Event 1 handheld FTIR data.

| **Label** | **Actual Sex** | **Predicted as F** | **Predicted as M** | **% Correct** | **ID Correct (Y/N)?** | **Label** | **Actual Sex** | **Predicted as F** | **Predicted as M** | **% Correct** | **ID Correct (Y/N)?** |
| --- | --- | --- | --- | --- | --- | --- | --- | --- | --- | --- | --- |
| HH01 | M | 5 | 0 | 0 | N | HH12 | M | 0 | 5 | 100 | Y |
| HH02 | M | 0 | 5 | 100 | Y | HH13 | M | 0 | 5 | 100 | Y |
| HH03 | M | 0 | 5 | 100 | Y | HH14 | M | 0 | 5 | 100 | Y |
| HH04 | M | 0 | 5 | 100 | Y | HH15 | F | 4 | 1 | 80 | Y |
| HH05 | M | 0 | 5 | 100 | Y | HH16 | F | 5 | 0 | 100 | Y |
| HH06 | M | 1 | 4 | 80 | Y | HH17 | F | 5 | 0 | 100 | Y |
| HH07 | M | 0 | 5 | 100 | Y | HH18 | F | 5 | 0 | 100 | Y |
| HH08 | M | 0 | 5 | 100 | Y | HH20 | F | 5 | 0 | 100 | Y |
| HH09 | M | 1 | 4 | 80 | Y | HH21 | F | 5 | 0 | 100 | Y |
| HH10 | M | 0 | 5 | 100 | Y | HH22 | F | 5 | 0 | 100 | Y |
| HH11 | M | 0 | 5 | 100 | Y | HH24 | F | 5 | 0 | 100 | Y |
